# Fluctuations in T cell receptor and pMHC interactions regulate T cell activation

**DOI:** 10.1098/rsif.2021.0589

**Published:** 2022-02-09

**Authors:** Joseph R. Egan, Enas Abu-Shah, Omer Dushek, Tim Elliott, Ben D. MacArthur

**Affiliations:** ^1^ Mathematical Sciences, Stem Cells and Regeneration, University of Southampton, Southampton SO17 1BJ, UK; ^2^ Institute for Life Sciences, Stem Cells and Regeneration, University of Southampton, Southampton SO17 1BJ, UK; ^3^ Centre for Human Development, Stem Cells and Regeneration, University of Southampton, Southampton SO17 1BJ, UK; ^4^ Centre for Cancer Immunology, University Hospital Southampton, Southampton SO16 6YD, UK; ^5^ Sir William Dunn School of Pathology, University of Oxford, Oxford OX1 3RE, UK; ^6^ Kennedy Institute of Rheumatology, University of Oxford, Oxford OX3 7FY, UK; ^7^ Nuffield Department of Medicine, University of Oxford, Oxford OX3 7BN, UK; ^8^ Alan Turing Institute, London NW1 2DB, UK

**Keywords:** T cell activation, mathematical model, stochastic processes, information theory, immunology

## Abstract

Adaptive immune responses depend on interactions between T cell receptors (TCRs) and peptide major histocompatibility complex (pMHC) ligands located on the surface of T cells and antigen presenting cells (APCs), respectively. As TCRs and pMHCs are often only present at low copy numbers their interactions are inherently stochastic, yet the role of stochastic fluctuations on T cell function is unclear. Here, we introduce a minimal stochastic model of T cell activation that accounts for serial TCR-pMHC engagement, reversible TCR conformational change and TCR aggregation. Analysis of this model indicates that it is not the strength of binding between the T cell and the APC cell *per se* that elicits an immune response, but rather the information imparted to the T cell from the encounter, as assessed by the entropy rate of the TCR-pMHC binding dynamics. This view provides an information-theoretic interpretation of T cell activation that explains a range of experimental observations. Based on this analysis, we propose that effective T cell therapeutics may be enhanced by optimizing the inherent stochasticity of TCR-pMHC binding dynamics.

## 1. Introduction

Lymphocytes are responsible for immunity and a subset known as T cells are critical for adaptive immunity [1]. T cell receptors (TCRs) located on the T cell surface reversibly bind to peptide major histocompatibility complex (pMHC) ligands located on the surface of antigen presenting cells (APCs) [2]. This interaction can generate a signalling cascade within the T cell [3], leading to a variety of functional responses [4], including the production of soluble messengers called cytokines [5]. Furthermore, an activated T cell is stimulated to proliferate, thereby generating progeny that can differentiate into effector cells [3]. These mature T cells are then able to clear antigen from the body by seeking out and destroying harmful pathogen-infected or tumour cells [6]. Yet despite decades of research, it is still unclear which TCR proximal mechanisms are primarily responsible for transmitting the information encoded in the pMHC ligand to the T cell intracellular signalling pathways [7–14].

Each TCR has a short intracellular domain that, alone, does not have the capacity to initiate signalling [1]. Consequently, a TCR associates with three CD3 subunits to facilitate signal transduction to the T cell interior [15]. The CD3 subunits have tails extending into the cytoplasm that contain multiple copies of the immuno-receptor tyrosine activation motif (ITAM) [3]. The phosphorylation of ITAMs is considered one of the earliest events in the signalling cascade that leads to T cell activation [4]. Kinases, such as LCK, are molecules that phosphorylate ITAMs and therefore favour signalling. By contrast, phosphatases, such as CD45, are molecules that dephosphorylate ITAMs and therefore inhibit signalling.

Three or four main mechanisms have been proposed to initiate signalling events following pMHC-TCR binding [2,6,14–18], all of which are likely to shift the balance in favour of ITAM phosphorylation [15]. One mechanism is the segregation of CD45 molecules from the TCR-CD3 complex [16] that could allow for the stable phosphorylation of ITAMs by LCK. A second mechanism is the aggregation of TCR-CD3 complexes and their subsequent ‘microcluster’ formation [19–21] that could increase the proximity of LCK molecules, leading to enhanced ITAM phosphorylation. A third mechanism is a physical and/or chemical change (generally referred to as a conformational change [7,8,12,22,23]) in the TCR-CD3 complex, possibly in the cytoplasmic tails that could expose their ITAMs to enhanced phosphorylation. A fourth mechanism [14] (which is arguably a sub-mechanism of the third mechanism [15]) is the generation of forces tangential to the T cell surface caused by the movement of T cells as they scan the surface of APCs for antigenic peptides [13]. These mechanical forces, such as pulling or shearing, could lead to the uncoupling of the CD3 tails from the T cell membrane, exposing their ITAMs to phosphorylation by LCK.

**Figure 1.**
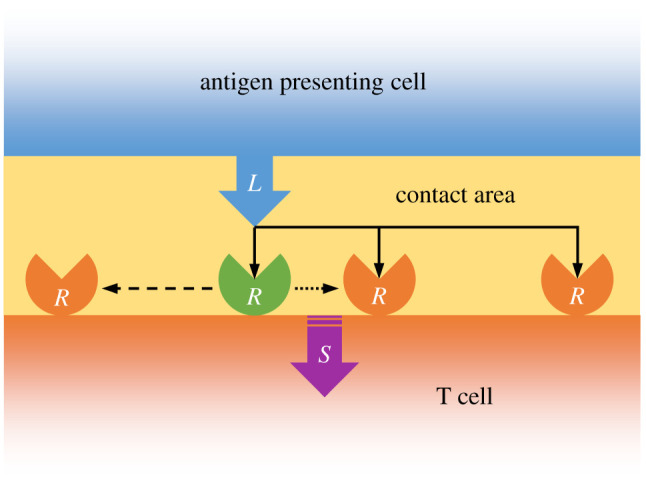
Schematic of the three modelled mechanisms involved in TCR-pMHC binding. (1) Solid black arrows represent a pMHC ligand, *L,* serially engaging with multiple TCRs, *R,* within the contact area. (2) The green TCR represents a conformational change upon pMHC ligand binding. The dashed black arrow represents the TCR reverting back to its original state at some time after unbinding. (3) The dotted black arrow represents TCR aggregation following pMHC ligand binding. The combination of these three mechanisms generates a signal, *S,* within the T cell.

It is likely that not one mechanism alone is responsible for the initiation of signalling events [24]. For example, it has been proposed that mechanical forces induce conformational changes [2,13–15,25] which subsequently induce aggregation and clustering [15,17]. Others have advocated that conformational change is instead directly induced by pMHC ligand binding [26,27] and is reversible [7,14,18,22,28]. It has also been argued that both conformational TCR change and TCR clustering are necessary for T cell activation [8] and may improve antigen discrimination [29,30]. Although some have argued that it is unnecessary [18,31,32], others have supported the view that signalling requires just a few pMHC ligands to serially bind multiple TCRs [33–36] and that this serial engagement could lead to a conformational change in each TCR [37,38]. The three mechanisms of serial TCR-pMHC engagement, reversible TCR conformational change and TCR aggregation are shown schematically in figure 1. Notably, it has been suggested that a combination of these three mechanisms may allow the T cell to efficiently scan the APC surface with high specificity and sensitivity for rare pMHC ligands presented at low copy numbers [1,4,10,15,33,36,39].

Indeed, there is increasing evidence that T cell activation can be induced by as few as approximately 1–10 pMHC ligands [31,34,36,40–42] and that microclusters may contain as few as approximately 10–100 TCRs [19–21, 33,34,43]. At such low copy numbers the TCR-pMHC binding dynamics are inherently stochastic, yet the effect of this stochasticity on T cell activation is unclear. Stochastic fluctuations have been shown to be functionally important in numerous other biological contexts [44–46]; therefore, it is conceivable that the T cell has evolved to use these fluctuations to enhance its own function.

Here, we develop a minimal stochastic model of the TCR-pMHC binding dynamics that includes serial TCR-pMHC engagement, reversible TCR conformational change and TCR aggregation. We show that, collectively, these three mechanisms are both necessary and sufficient for the T cell to convert stochastic fluctuations in the TCR-pMHC binding dynamics into a well-defined signal. Based on this analysis, we propose that the T cell response to an APC is not determined by the strength of TCR-pMHC binding *per se*, but rather by the information conveyed to the T cell by the encounter, as assessed by the entropy rate of the TCR-pMHC binding dynamics. We validate this hypothesis against a range of experimental studies, including a number of dose–response datasets, before discussing the implications for T cell based therapeutics.

## 2. Results

### 2.1. Fluctuations in TCR-pMHC binding dynamics generate information

To start, we will introduce some information-theoretic notions in the context of a simple model of TCR-pMHC binding, before discussing how they apply to a more realistic model of T cell activation.

Consider the process of TCR-pMHC reversible binding, given by the following reactions:

2.1 $$L+R\overset{k_{\mathrm{on}}}{\underset{k_{\mathrm{off}}}{⇋}}\;B,$$

where *L* denotes the pMHC ligand, *R* denotes the TCR, *B* denotes the TCR-pMHC complex, *k*_off_ is the rate of unbinding, *k*_on_/*ν* is the rate of binding [47] and *ν* is the two-dimensional (2D) contact area in which the biochemical reactions take place.

At low copy numbers, these reactions will be inherently stochastic and the copy number of the TCR-pMHC complex will accordingly fluctuate randomly over time. To quantify the extent of this stochasticity, we will use two measures. First, the Shannon entropy, *H*(*B*) (in bits), given by

2.2 $$H(B)=-\sum_{i=0}^{B_{\max}}p(i)\log_{2}p(i),$$

where *B*_max_ is the maximum number of TCR-pMHC complexes (given by equation (4.1) in the Material and methods) and *p*(*i*) is the stationary probability that *i* copies of the TCR-pMHC complex are present (given by equation (4.3) in the Material and methods). In what follows, we will assume that the T cell responds on a slower time scale than the TCR-pMHC binding dynamics, and consider properties of stationary probability distributions only. In general, the Shannon entropy is a simple measure of information or ‘disorder’ [48]. In the context of the T cell–APC contact area, it is the average amount of information imparted to the T cell per TCR-pMHC binding/unbinding event. As such, although it is a useful measure of information, the Shannon entropy does not take account of the speed of the underlying reactions, which will vary with the kinetic rate parameters. Therefore, the Shannon entropy cannot distinguish between fast and slow dynamics.

To clarify this distinction, we will use an alternative measure: the entropy rate, *H*′(*B*) (in bits per second), which is calculated as the mean reaction rate (i.e. the average number of binding/unbinding events per second) multiplied by the Shannon entropy. For the reversible binding reactions given in equation (2.1), the entropy rate is

2.3 $$H^{\prime }(B)=2k_{\mathrm{off}}\langle B\rangle H(B),$$

where 〈*B*〉 is the mean of the TCR-pMHC complex stationary probability distribution (for details see §2.2 of the electronic supplementary material). In the context of the T cell–APC contact area, the entropy rate is the average amount of information imparted to the T cell by the TCR-pMHC binding dynamics per second. Therefore, unlike the Shannon entropy, the entropy rate can distinguish between fast and slow dynamics.

While it has a useful information-theoretic interpretation, the entropy rate is complex to calculate in practice. However, we can similarly define the ‘variance rate’, ${\text{Var}}^{\prime }(B)$, as

2.4 $${\text{Var}}^{\prime }(B)=2k_{\mathrm{off}}\langle B\rangle \text{Var}(B),$$

where Var(B) is the variance of the TCR-pMHC complex stationary probability distribution. Although the variance rate does not have an information-theoretic interpretation, it exhibits similar features to the entropy rate for the simple dynamics described here and is more analytically tractable (for details, see §§2.3 and 4.2 of the electronic supplementary material). We will make use of this connection in the next section, where we analyse a more realistic model of TCR-pMHC binding dynamics.

**Figure 2.**
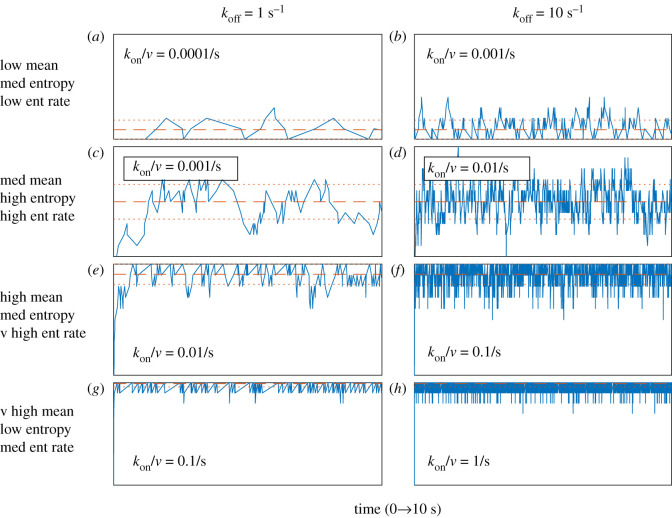
Fluctuations in TCR-pMHC dynamics generate information. Blue lines show representative stochastic simulations of the TCR-pMHC complex copy number, *B,* for the first 10 s of the reversible binding reactions given in equation (2.1). Dashed red lines show the mean and dotted red lines show the mean plus/minus one standard deviation. In all panels, the number of TCRs, *R*_max_ = 10, and the number of pMHC ligands, *L*_max_ = 1000, which gives the maximum number of TCR-pMHC complexes, *B*_max_ = 10, via equation (4.1). The binding rate, *k*_on_/*ν,* and unbinding rate, *k*_off_, are varied over orders of magnitude within a plausible physiological range, as described in the Material and methods.

To illustrate these concepts, figure 2 shows some representative stochastic simulations of TCR-pMHC reversible binding using Gillespie’s direct method [49,50]. Note that a relatively high number of pMHC ligands (1000) has been used in figure 2 to highlight the differences between the mean, Shannon entropy and entropy rate. Lower numbers of pMHC ligands (as low as 1) are considered in the following section. Three features of these simulations are notable.

First, while the mean number of TCR-pMHC complexes increases monotonically with the binding rate (*k*_on_/*ν*), both the Shannon entropy and the entropy rate initially increase as the binding rate increases, but then decrease as the binding rate increases further still (to see this compare the panels in each column of figure 2). This biphasic pattern occurs because fluctuations are minimal when binding is very weak or very strong (i.e. when complexes do not easily associate or dissociate, respectively), yet become larger at intermediate affinities that allow both binding and unbinding events to easily occur.

Second, while the mean number of TCR-pMHC complexes and Shannon entropy are dependent on three model parameters: the total number of pMHC ligands and TCRs at the contact area (which we denote *L*_max_ and *R*_max_, respectively) and the 2D dissociation constant, *K*_d_, given by

2.5 $$K_{d}=\frac{\nu k_{\mathrm{off}}}{k_{\mathrm{on}}};$$

the entropy rate (given by equation (2.3)) is explicitly dependent on both the binding rate and the unbinding rate (rather than simply the ratio of the two, *K*_d_). Thus, dynamics associated with different kinetic rate parameters may have the same mean number of TCR-pMHC complexes and Shannon entropy, but very different entropy rates (to see this compare the panels in each row of figure 2; in each case, the entropy rate in the right column is an order of magnitude higher than that in the left column).

Third, for a fixed unbinding rate, the maximum entropy rate (and therefore the maximum rate at which information can be imparted to the T cell) is achieved via a trade-off between the average number of TCR-pMHC complexes and the average magnitude of the stochastic fluctuations. So, the TCR-pMHC binding dynamics illustrated in figure 2*f* have the largest entropy rate of all the panels because they combine both a relatively high mean with a relatively high Shannon entropy.

Collectively, this reasoning suggests that fluctuations in TCR-pMHC binding dynamics can generate information and thereby may have an important, but as yet unexplored, part to play in regulating T cell activation.

### 2.2. TCR-pMHC fluctuations regulate T cell activation

To investigate this possibility further, we sought to construct a minimal model of the TCR-pMHC binding dynamics that includes the effects of serial TCR engagement, reversible TCR conformational change and TCR aggregation. Our minimal model (which is referred to as ‘model 1’ in §5.1 of the electronic supplementary material) consists of the following set of reactions:

2.6 $$L+R_{I}\overset{k_{\mathrm{on}}}{⟶}B,$$

2.7 $$B\overset{k_{\mathrm{off}}}{⟶}L+R_{A},$$

2.8 $$L+R_{A}\overset{k_{\mathrm{on}}}{⟶}B,$$

2.9 $$R_{A}\overset{k_{\mathrm{off}}}{⟶}R_{I}$$

2.10 $$\mathrm{and}\;\;R_{A}+B\overset{k_{\mathrm{off}}}{⟶}R_{A}+B+S,\;$$

where *R*_I_ and *R*_A_ denote ‘inactive’ TCRs and ‘active’ TCRs, respectively, and *S* denotes an activating T cell signal. Note that an active TCR can be interpreted as one that has undergone a conformational change owing to pMHC ligand binding and the generation of a signal can be interpreted as a subsequent consequence of TCR aggregation; see figure 1. We emphasize that equations (2.6)–(2.10) are not meant to be a detailed model of every aspect of TCR-pMHC binding and T cell activation. Rather, they encapsulate key mechanisms in a parsimonious way that allows for a transparent exploration of their consequences. Particularly, equation (2.10) captures salient features of TCR aggregation without recourse to relatively complex stochastic reaction–diffusion processes, which, although more mechanistically detailed, may be less tractable and harder to interpret. A more detailed explanation of how each reaction relates to each of the three TCR proximal mechanisms detailed in figure 1 is provided in the Material and methods.

This modelling framework is useful because it accounts for additional mechanisms of importance, yet central aspects of the reversible binding reactions given in equation (2.1) are conserved (for details, see §5 of the electronic supplementary material). In particular, the dynamics of the TCR-pMHC complex number, *B*, pMHC ligand number, *L*, and the sum of the inactive and active TCR numbers, *R* = *R*_I_ + *R*_A_, are equivalent to those of the straightforward reversible binding reactions. Thus, calculations of the mean number of TCR-pMHC complexes, Shannon entropy and the variance/entropy rates described in the previous section also apply to this model.

**Figure 3.**
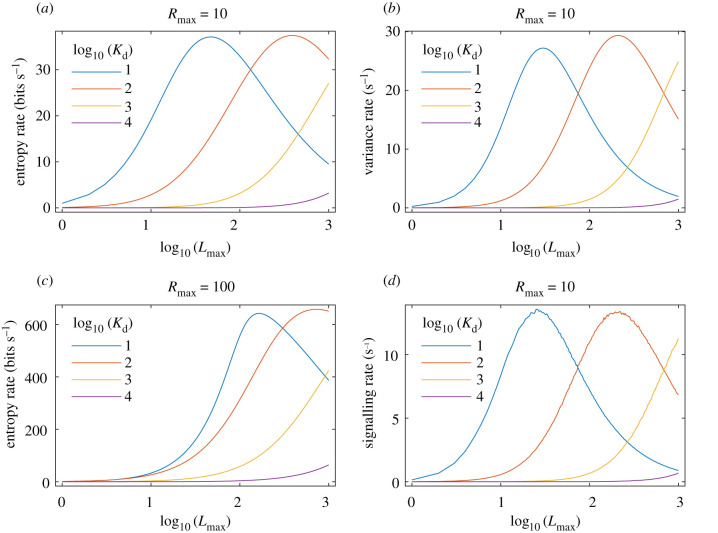
T cell signalling rate is regulated by TCR-pMHC fluctuations. Theoretical dose–response curves where dose on the *x*-axis is given by the number of pMHC ligands (*L*_max_) and the legend gives the 2D dissociation constant (*K*_d_). Response on the *y*-axis is given by the entropy rate (*a*,*c*) and variance rate (*b*) of the TCR-pMHC complex stationary probability distribution, and the mean signalling rate (*d*) based on the minimal model of T cell activation as calculated via stochastic simulations of the reactions given in equations (2.6)–(2.10). Comparison of (*a*) and (*c*) shows that variation in the number of TCRs (*R*_max_) does not affect the qualitative nature of the curves. In all panels *k*_off_ = 1/*s*. See Material and methods for justification of these and other literature-derived parameter values.

Moreover, the effects of these quantities on signal generation may now be explored. In §5.1 of the electronic supplementary material, we show that the mean number of active TCRs, 〈*R*_A_〉, is equal to the variance of the TCR-pMHC complex number, Var(B), in wide regions of parameter space. This is notable because in this framework a T cell signal is stochastically generated if an active TCR is in close proximity to a TCR-pMHC complex (see equation (2.10)). Consequently, this implies that the mean signalling rate, $\left\langle \dot{S}\right\rangle$ (i.e. the average rate at which a signal is generated), is approximately equal to half the variance rate, ${\text{Var}}^{\prime }(B)$, for a wide range of parameter values, as shown in figure 3. This reasoning suggests that serial TCR-pMHC engagement, reversible TCR conformational change and TCR aggregation work collectively to allow the T cell to process environmental information appropriately.

In addition to offering an explanation of why these mechanisms are central to T cell activation, this perspective has important implications for optimization of the T cell response. In this minimal model, increasing the TCR-pMHC binding rate or the number of pMHC ligands (i.e. dose) initially increases the variance of the TCR-pMHC complex number and thereby the mean number of active TCRs and mean signalling rate (starting from a low binding rate or low dose). However, as the binding rate or dose increases further still, the variance of the TCR-pMHC complex number will fall and, while the mean number of TCR-pMHC complexes will continue to increase, the mean number of active TCRs will start to decrease. This, in turn, leads to a decrease in the mean signalling rate. Thus, because T cell activation is not regulated solely by the binding strength (i.e. affinity) between the TCR and pMHC molecules, but also by their dynamic fluctuations, maximal T cell activation is predicted to occur at an intermediate affinity (particularly with an intermediate to high physiological dose) or an intermediate dose (particularly with an intermediate to high physiological affinity). Figure 3 shows results of the binding and activation dynamics that illustrate these points, which are discussed further in the following section.

A similar alternative minimal model (referred to as ‘model 2’ in §5.2 of the electronic supplementary material) gives a mean signalling rate that is equivalent to that shown in figure 3*d* over a wide area of parameter space. This suggests that the minimal model described by equations (2.6)–(2.10) is representative of a wider class of models that exhibit shared characteristics. Two key characteristics that are common to both minimal models are that (i) TCRs are initially inactive and can become active following pMHC ligand binding and (ii) the generation of a signal requires an unbound TCR and a bound TCR, one of which must be in an active state. A natural consequence of these shared characteristics is that a single pMHC ligand reversibly binding with a completely isolated TCR could not at first generate a signal based on either model. Such an event would not only allow the TCR to change from inactive to active (and vice versa) but also need to induce at least one further TCR to move sufficiently close to the original TCR before a signal could be generated. Furthermore, the generation of a signal requires at least one ligand in model 1 but at least two ligands in model 2. It is worth noting that there is recent experimental evidence in support of each of these scenarios [18,34,36].

Collectively, these results indicate that stochastic fluctuations, as quantified by the variance rate of the TCR-pMHC binding dynamics, may regulate T cell activation. While we could not obtain a corresponding analytical result for the entropy rate, the variance rate and entropy rate are very closely related and numerical simulations of TCR-pMHC binding dynamics indicate a similar dependency (cf. figure 3*a* and 3*b*). This suggests that the variance rate is an analytically convenient proxy for the more biologically meaningful entropy rate as a measure of the magnitude and rate of TCR-pMHC fluctuations.

### 2.3. Experimental validation of optimal affinity and optimal dose

As described above, our model suggests that intermediate affinity and intermediate dose scenarios can give rise to highly stochastic, information-rich, dynamics which the T cell is able to process, via simple molecular mechanisms, into a defined cellular response. To investigate the validity of this view, we sought to determine its experimental support.

First, a number of experimental studies have reported that the T cell response is maximized at an intermediate affinity [51–60]. Moreover, some of these studies have shown that an optimal affinity exists for both proximal and distal activation events (i.e. early and later T cell responses) [55–57].

**Figure 4.**
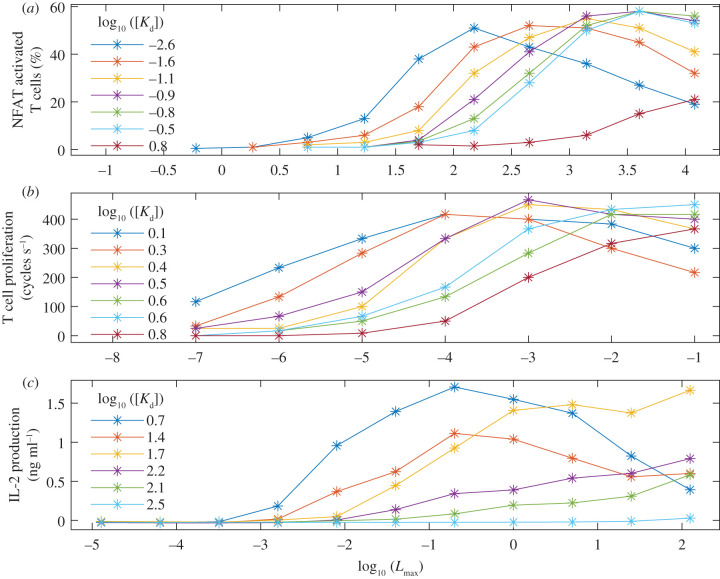
Experimental data are consistent with theory. Experimental dose–response curves (*a*) from [66] using A5 T cells expressing the 14.3.d TCR, (*b*) from [54] using T cells expressing an AH1-specific TCR and (*c*) using naive T cells transduced with the 1G4 TCR (see Material and methods). Dose on the *x*-axis is given by the concentration of (*a*) antibodies (in μm^−2^) and (*b*,*c*) peptide (in μM). The legend gives the 3D affinity (in μM). Response on the *y*-axis is given by (*a*) the percentage of T cells in which the transcription factor NFAT was activated, (*b*) the rate of T cell proliferation and (*c*) the density of the cytokine interleukin 2 (IL-2) that was produced.

Second, other experimental studies have reported that the T cell response is maximized at an intermediate dose for both early [61,62] and later [63–65] T cell responses, particularly for higher affinity ligands [54,66–69]. For example, figure 4*a*,*b* shows dose–response curves from two previous studies [54,66] and figure 4*c* shows previously unpublished experimental dose–response data. Briefly, all of these studies used a TCR and varied the affinity via a panel of ligands [54,66]. In the earlier study, the ligands were antibodies [66] that were immobilized on micro-titre plates before being stimulated by T cells. In the later study and here, the ligands were altered peptide ligands that were either directly added to cultures containing T cells [54] or pulsed into monocyte-derived dendritic cells before being co-cultured with T cells (see Material and methods). Despite the experimental variation, all of the panels of figure 4 are qualitatively consistent with our model predictions shown in figure 3. Furthermore, all of these datasets exhibit similar features regardless of the T cell responses that were measured or the experimental techniques that were implemented.

Third, Lever *et al.* [67] summarized the results of their extensive dose–response experiments (some of which have been repeated [70]) in the following four statements:

1. Dose–response curves are bell-shaped for high- but not low-affinity pMHC ligands.
2. The peak amplitude of bell-shaped dose–response curves is independent of affinity.
3. A single intermediate affinity pMHC ligand produces largest response at low pMHC doses.
4. Different intermediate affinity pMHC ligands produce the largest response at high pMHC doses.

Statements 1, 2 and 4 are consistent with our model predictions shown in figure 3, as well as being broadly consistent with the other experimental dose–response datasets shown in figure 4. However, this is not the case for statement 3, where our theory and the other dose–response datasets show that the pMHC ligand with the highest physiological affinity produces the largest response at low doses. This discrepancy might be explained by the affinity-enhanced 1G4 TCR used in the Lever *et al.* [67] (and subsequent [70]) study that extends to very high supra-physiological affinities.

A quantitative fit of our model to the experimental data would have allowed for a more robust validation of the model. However, the data in figure 4 report three-dimensional (3D) affinities that were measured via techniques such as surface plasmon resonance. By contrast, our model is parametrized by 2D affinities measured via the adhesion frequency assay technique [33] (see Material and methods). Although there have been efforts to directly convert the 3D kinetics to their 2D counterparts [71–74] it has been suggested that simple conversions may not be possible [10]. On the other hand, 2D affinity has been shown to correlate positively with 3D affinity, despite their respective kinetics not exhibiting such positive correlations [33,75]. As such, we felt that a qualitative comparison between the model and the available experimental data was more appropriate at this stage.

Collectively, these results indicate that simple information-theoretic reasoning can help interpret complex dose–response data, and suggest that the T cell response is regulated by TCR-pMHC fluctuations.

## 3. Discussion

A general communication system consists of at least three interconnected parts: an information source, a channel and a destination [76]. In the context of T cell activation, TCR-pMHC binding dynamics can be thought of as the information source; intracellular signalling pathways as the channel; and the cell nucleus as the destination. From this perspective, stochasticity in TCR-pMHC binding dynamics generates a ‘message’ which, depending on the kinetic rate parameters, may contain more or less information. Moreover, the average information content per second of this message, as assessed by the entropy rate, represents the average rate at which peptide-specific information is conveyed to the nucleus via signalling pathways. Based on this reasoning, we propose that T cell activation is regulated by the entropy rate of the TCR-pMHC binding dynamics. More generally, this reasoning suggests that tools from information theory may help to shed light on the complex information-processing mechanisms involved in T cell activation.

Indeed, a study by Ganti *et al.* [77] focused on channel capacity via a relatively complex model of the T cell signalling pathway. This is in contrast to our approach where we have focused on the information source via a relatively simple model of the pMHC-TCR binding dynamics. Ganti *et al.* [77] also advocate kinetic proof-reading whereby pMHC ligands are required to remain bound to TCRs for a sufficiently long time in order to initiate T cell signalling. Although the model developed here has no requirement for a minimum duration of engagement (or ‘dwell-time’ [10,51]), the bio-chemical reactions of kinetic proof-reading (e.g. [5]) and reversible conformational change (equations (2.6)–(2.9) and §5 of the electronic supplementary material) are similar in that both require a TCR to undergo at least one additional transition to an active (or signalling-competent [5]) state following initial TCR-pMHC binding before a signal can be generated.

A previous stochastic model of TCR-pMHC reversible binding [78] similarly advocated that the initiation of T cell signalling required a given number of TCR-pMHC complexes to remain bound for a given minimum dwell-time. By contrast, we posit that faster kinetics can potentially be advantageous to the T cell by increasing the entropy/signalling rate (cf. the left and right columns of panels in figure 2). Consequently, our model is inherently incompatible with the concept of TCR-pMHC dwell-time, which posits that slow unbinding rates (supported by 3D kinetics [79] and more recent optogenetic approaches [61,80]) are necessary for T cell activation. Instead, our stochastic model is consistent with the ‘fast kinetics based serial engagement model’ [1,33,35], which (like our study) is informed by 2D kinetics. Furthermore, our model is in broad agreement with two recent studies that observed a temporal sequence of short-lived pMHC-TCR binding events [36,81] that were ‘sufficiently close in space’ [81] or required ‘two or more TCRs within a range of 20 nm’ [36]. Note that in equation (2.10) an activating signal is, indeed, generated by two TCRs (one bound and one unbound) that are in close spatial proximity to each other.

Our model is also arguably in accordance with the ‘sustained signalling model’ [5,53,82] in that, here, TCRs remain active for a period of time after unbinding, during which they can contribute to signalling, before reverting back to their inactive (or ‘basal’ [82]) state. Moreover, this feature of our model could provide the ‘memory’ that has been suggested as necessary for conformational change models to be compatible with ‘confinement time models’ [83,84]. In addition, our model is consistent with the ‘integrated TCR triggering model’ [15] in which TCR-pMHC binding leads to segregation of the TCR-CD3 complex from phosphatases, as well as conformational change and aggregation in the TCR-CD3 cytoplasmic tails. Thus, although we do not explicitly model phosphatase segregation, by similarly assuming that such segregation occurs upon TCR-pMHC binding, our model is arguably compatible with the ‘kinetic-segregation model’ [16,85,86].

It is noteworthy that, in addition to the TCR-related datasets described in the Results, bell-shaped dose–responses have also been experimentally observed for chimeric antigen receptors (CARs) [60,68,87,88]. If our model was applicable to both TCRs and CARs then the proposed reversible conformational change might be expected to occur in the intracellular signalling domains (such as the CD3 tails) that are common to both receptors. Recent CAR experiments not only support such ligand-binding-induced conformational changes but also support subsequent CAR aggregation [88] (i.e. a further requirement of our model).

Previous studies have argued that such bell-shaped dose–responses can be explained by TCR cross-linking [89], a TCR-proximal negative feedback loop [90], a TCR/CAR-proximal incoherent feed-forward loop [67,68], TCR/CAR down-regulation [69,91] or CAR dimerization [88]. Such minimal models were largely based on a deterministic framework that accounts for average copy numbers but not their fluctuations. Although a deterministic model of serial TCR-pMHC engagement, reversible TCR conformational change and TCR aggregation gives a signalling rate similar to that shown in figure 3*d* (see §5 of the electronic supplementary material), only by taking a stochastic view have we been able to provide an information-theoretic explanation for why these three mechanisms might be utilized by the T cell.

One of the most challenging features of studying TCR signalling is the ample counter-examples for any given model of T cell activation. As described above, there are numerous models that have attempted to explain the relationship between affinity and activation, and experimental exceptions abound for each model [23]. For example, there are examples of relatively high-affinity ligands that do not efficiently signal at any concentration [92–94], ligands that are low affinity yet extremely sensitive for antigen recognition [95] and ligand sets with very large differences in activation despite smaller changes in affinity [96]. Arguably, some of these observations can be captured by our model since it is dependent on both the 2D unbinding and binding rates, rather than the affinity alone. However, given that there are now at least six different mathematical models that predict bell-shaped dose–responses, a future study is arguably warranted to provide an extensive comparison of the models.

To help discriminate between these models such a study would benefit from parallel experiments that combine dose–response assays with adhesion frequency assays. However, a positive correlation between the potency (i.e the dose which gives a half-maximal response) and the dissociation constant has been shown to exist for multiple mathematical models of T cell activation [5,97]; therefore, it would be important to consider the entire dose–response curve (as has been performed here and previously [67]) rather than a single summary measure alone [33]. Such experiments would also allow for an improved quantitative validation of our model. For example, they could help to confirm whether the relatively large difference between the maximum of the red and yellow/blue curves in figure 4*c* was due to differences in the 2D unbinding rates of two pMHC ligands (as predicted by our model) given that they had similar 3D affinities and their stability was found to be comparable [98]. In addition, such experiments would help to determine the validity of applying our model to both up-stream and down-stream functional readouts for a given pMHC ligand.

The implications of these considerations are perhaps most important for designing the next generation of immunotherapies. For example, identifying the optimal affinity and dose is central to the design of CAR T cell therapies [57–60,68,91,99–102] as well as cancer vaccines [54–57,64]. Our information-theoretic approach provides a framework to guide the optimization of the T cell response via modification of the affinity or dose of the TCR-pMHC binding dynamics. This issue is considered further in §2.4 of the electronic supplementary material, where we provide a numerical procedure to calculate the optimal affinity under conditions in which the total numbers of both TCRs and pMHC ligands are fixed. If it were possible to manipulate both the binding and unbinding rates then our analysis suggests that the T cell response will increase with faster kinetics, providing that the optimal affinity is maintained.

Our results also provide a note of caution. Shannon’s seminal information theorems [76] show that it is unproductive for the entropy rate of a message to exceed the communication system’s channel capacity, because the channel capacity sets an upper limit to the rate of error-free information transmission. This suggests that there is a limit to the rate at which the T cell can process information, which is set by the intracellular signalling pathways that transmit signals from the cell surface to the nucleus. Thus, there may be a limit to our ability to engineer T cell therapeutics based on manipulation of the TCR-pMHC kinetic rate parameters, unless the capacity of the signalling pathway(s) that transmit these messages can also somehow be increased. To quote Lombardi *et al.* [103]: ‘[t]he goal in the field of communication engineering is to optimize the transference of information through channels conveniently designed’. We speculate that the same may be true for T cell engineering.

Although we have focused on the T cell response, the simplicity of our model means that an information-theoretic perspective of receptor–ligand binding could have application to a wide range of other therapeutics. For instance, experimental evidence for binding-induced conformational change that subsequently induces aggregation and clustering not only exists for the TCR [104] but has also been found for the B cell receptor [105,106].

## 4. Material and methods

### 4.1. Fluctuations in the TCR-pMHC binding dynamics

In the context of the reactions of equation (2.1), let *B*_max_ and *U*_max_ denote the smaller and larger, respectively, of the total number of pMHC ligands, *L*_max_, and total number of TCRs, *R*_max_, given by

4.1 $$B_{\max}=\text{min}(L_{\max},R_{\max})$$

and

4.2 $$U_{\max}=\max(L_{\max},R_{\max}).$$

Note that *B*_max_ is also the maximum number of TCR-pMHC complexes. The stationary probability distribution of the TCR-pMHC complex number, *p*(*B*), is given by

4.3 $$p(B;B_{\max},U_{\max},K_{d})=\frac{a(B;B_{\max},U_{\max},K_{d})}{Z(B_{\max},U_{\max},K_{d})},$$

where

4.4 $$a(B;B_{\max},U_{\max},K_{d})=\left(\begin{array}{c} B_{\max}\\ B \end{array}\right)\left(\begin{array}{c} U_{\max}\\ B \end{array}\right){K_{d}}^{-B}B!$$

and

4.5 $$Z(B_{\max},U_{\max},K_{d})=\sum_{i=0}^{B_{\max}}a(i;B_{\max},U_{\max},K_{d}),$$

and where *K*_d_ is given by equation (2.5). The probability of there being at least one TCR-pMHC complex in the contact area, *P*_a_, is given by

4.6 $$P_{a}=1-\frac{1}{Z(B_{\max},U_{\max},K_{d})}.$$

Equation (4.6) is commonly referred to as the ‘probability of adhesion’ between two cells [33]. Full details of these and further calculations are provided in the electronic supplementary material.

### 4.2. Model parametrization

To produce figures 2 and 3, we used parameters from the literature. Specifically, Huang *et al.* [33] performed a series of adhesion frequency assays in which a T cell was mechanically brought in and out of contact with an APC for varying durations on multiple occasions. An approximation to the probability of adhesion, *P*_a_, given by equation (4.6) (and its time-dependent generalization) was fitted to the proportion of contacts that had resulted in adhesion. Table 1 summarizes the key parameters from the Huang *et al.* study, which allows for conversion to the parameters described in this study (i.e. *L*_max_, *R*_max_, *K*_d_ and *k*_on_/*ν*). Combining table 1 with equation (2.5) gives an order of magnitude range of *K*_d_ ∈ [10^1^, 10^4^] and *k*_on_/*ν* ∈ [10^−4^, 10^0^] s^−1^. Table 1 also gives an order of magnitude range of *L*_max_ ∈ [10^0^, 10^2^]. We extended the upper limit of *L*_max_ by an order of magnitude because many dose–response studies consider a wider range of doses (e.g. [67]). Finally, table 1 gives a parameter estimate of *R*_max_ ∼ 10^1^. Other studies have found that the number of TCRs in individual microclusters is approximately 10–100 [19–21,33,34]. Therefore, we also considered *R*_max_ ∼ 10^2^ in figure 3*c* as a sensitivity analysis. Note that figure 2 characterizes the model for fixed values of *L*_max_ and *R*_max_, and with varying values of *k*_on_/*ν* and *k*_off_. By contrast, figure 3 characterizes the model for fixed values of *k*_off_, and with varying values of *L*_max_, *R*_max_ and *k*_on_/*ν*.

**Table 1.** Parameters derived from Huang *et al.* [33] based on experiments performed at 37°C. *Estimation* refers to whether the parameter was directly measured or fitted from data. *Name* is how the parameter was described in the Huang *et al.* study. *Notation* denotes the parameter based on the notation in this study. *Value* gives an order of magnitude estimate or range. Note that the *2D contact area* was described as ‘a few percent’ of 3 μm^2^ or 1 μm^2^ depending on the type of apparatus used in the experiments.

estimation	name	notation	value	units
measured	2D contact area	*ν*	10^−1^	μm^2^
measured	TCR density	*R*_max_/*ν*	10^2^	μm^−2^
measured	pMHC density	*L*_max_/*ν*	[10^1^, 10^3^]	μm^−2^
fitted	effective 2D affinity	*ν*^2^/*K*_d_	[10^−3^, 10^−6^]	μm^4^
fitted	2D off rate	*k*_off_	[10^0^, 10^1^]	s^−1^

### 4.3. Minimal model of signal generation

Equations (2.6), (2.7), (2.8) and (2.9) model a combination of the serial TCR-pMHC engagement and TCR reversible conformational change mechanisms. Specifically, equation (2.6) models an inactive TCR (i.e. a TCR in its resting state), *R*_I_, binding with a pMHC ligand, *L,* to form a TCR-pMHC complex, *B*. Equation (2.7) models a TCR conformational change whereby an inactive TCR enters an active state, *R*_A_, upon unbinding from the TCR-pMHC complex. For simplicity, we assume that an active TCR can bind with a pMHC ligand at the same rate as an inactive TCR, as shown by equation (2.8). Furthermore, equation (2.9) models an active TCR reverting to an inactive TCR. Equation (2.10) models the TCR aggregation mechanism whereby a signal, *S,* is generated, providing that an active TCR is in sufficient proximity to a TCR-pMHC complex.

The mean signalling rate shown in figure 3*d* was calculated via repeated stochastic simulations of the reactions given in equations (2.6)–(2.10) as follows. Initial conditions were: *R*_I_(0) = *R*_max_ = 10 and *R*_A_(0) = *B*(0) = *S*(0) = 0. The unbinding rate, *k*_off_, was fixed at 1/s and the binding rate, *k*_on_/*ν*, was varied between 10^−4^/s and 10^−1^/s to give the values of *K*_d_ shown in the legend as calculated via equation (2.5). Each stochastic simulation was run until either *S*(*t*) > 10^4^ or *t* > 10^4^ s and then the signalling rate was calculated as $\dot{S}=S(t)/t$. The mean signalling rate, $\left\langle \dot{S}\right\rangle$, was then calculated as the mean of $\dot{S}$ over 10 simulations for each set of parameter values.

The deterministic solutions of equations (2.6)–(2.10) are provided in §5 of the electronic supplementary material. Moreover, the approximate deterministic signalling rate is shown to be equal to half the approximate variance rate divided by the contact area. Therefore, interested readers may wish to approximate the model via the simple approximate variance rate provided in §4.2 of the electronic supplementary material or use the exact variance and entropy rates provided by equations (2.4) and (2.3), respectively. Matlab^®^ functions that provide robust computational calculations for the key equations have been made available at https://github.com/josephrobertegan/fluctuations.

### 4.4. T cell and monocyte preparation

The assay was performed as previously detailed [98,107]. In brief, human autologous T cells and monocytes were isolated from anonymized leukopoiesis products obtained from the UK National Health Service at Oxford University Hospitals (REC 11/H0711/7). Naive T cells were isolated using negative selection kits (Stemcell Technologies).

T cells were cultured at 37°C, $5\text{\%}\;{\mathrm{CO}}_{2}$, in RPMI 1640 (Roswell Park Memorial Institute) medium supplemented with 10% fetal bovine serum (Gibco), 5% penicillin–streptomycin (PenStrep, Gibco); 1× MEM non-essential amino acids solution, 20 mM HEPES, 1 mM sodium pyruvate, 2 mM Glutamax and 50 μM 2-mercaptoethanol (Sigma) (all from Thermo Fisher unless stated otherwise).

T cells were resuspended at 25 × 10^6^/ml in Opti-MEM serum-free medium containing mRNA for the 1G4 TCR*α*, TCR*β* and CD3*ζ* chains at 2 μg/10^6^ cells and electroporated at 300 V, 2 ms in an ECM 830 Square Wave Electroporation System (BTX).

Monocytes were enriched using a RosetteSep kit (Stemcell Technologies) and cultured at 1 − 2 × 10^6^/ml in 12-well plates with 1 ml of differentiation medium containing 50 ng ml^−1^ interleukin 4 (200–04 A, Peprotech) and 100 ng ml^−1^ granulocyte–monocyte colony-stimulating factor (11343125, Immunotools) for 24 h. For maturation, the following cytokines were added for an additional 24 h: 1 μM prostaglandin E_2_ (P6532, Sigma), 10 ng ml^−1^ interleukin 1β (201-LB-025/CF, Bio-Techne), 50 ng ml^−1^ tumour necrosis factor α (300-01A, Peprotech) and 20 ng ml^−1^ interferon γ (285-IF-100/CF, Bio-Techne).

### 4.5. T cell and monocyte co-culture

Monocyte-derived dendritic cells (moDCs) were pulsed with a titration of different variants of the NYE-ESO157–165 as in [98]. Loading was done for 1–2 h at 37°C. T cells and moDCs were mixed at a 1 : 1 ratio and incubated for 24 h before the supernatant was collected for downstream analysis.

### 4.6. ELISAs

Human interleukin 2 (IL-2) Ready-SET Go! ELISA kit (eBioscience/Invitrogen) and Nunc MaxiSorp 96-well plates (Thermo Fisher) were used according to the manufacturer’s instructions to test appropriately diluted (commonly fourfold) T cell supernatant for secretion of IL-2. The mean of three independent experiments is shown in figure 4*c*.

## Data accessibility

Supporting equations are provided in the electronic supplementary material [108]. Data and Matlab^®^ code for reproducing the figures in the paper are available at the following GitHub repository: https://github.com/josephrobertegan/fluctuations
